# Metformin Exerts Anti-inflammatory and Mucus Barrier Protective Effects by Enriching Akkermansia muciniphila in Mice With Ulcerative Colitis

**DOI:** 10.3389/fphar.2021.726707

**Published:** 2021-09-30

**Authors:** Haoran Ke, Fang Li, Wenlin Deng, Zitong Li, Siqi Wang, Pinjing Lv, Ye Chen

**Affiliations:** ^1^ Department of Gastroenterology, State Key Laboratory of Organ Failure Research, Guangdong Provincial Key Laboratory of Gastroenterology, Nanfang Hospital, Southern Medical University, Guangzhou, China; ^2^ Hainan General Hospital, Haikou, China; ^3^ Department of Pediatrics, The Sixth Affiliated Hospital of Sun Yat-sen University, Guangzhou, China

**Keywords:** intestinal barrier, inflammatory bowel diseases, probiotics, gut microbiota, mucin2

## Abstract

The present study aimed to determine if metformin exerts anti-inflammatory and mucus-protective effects *via* the gut microbiota. Metformin has extensive benefits including anti-inflammatory effects. Previous studies showed that metformin changed the gut microbiota composition and increases the number of goblet cells. Intestinal dysbiosis and goblet cell depletion are important features of ulcerative colitis (UC). The underlying mechanism and whether metformin can improve the mucus barrier in UC remain unclear. Metformin (400 mg/kg/day) was administered to mice with dextran sulfate sodium (DSS)-induced UC for 2 wk to investigate the effects of metformin on the intestinal mucus barrier. The gut microbiota was depleted, using antibiotics, to explore its role in the mucus-protecting effects of metformin. *Akkermansia muciniphila (A. muciniphila)*, which was enriched in metformin-treated mice, was administered to mice to investigate the effects of the bacteria on UC and the mucus barrier. Metformin attenuated DSS-induced UC in mice, as evidenced by the alleviation of diarrhea, hematochezia, and the decrease in body weight. The expression of mucin2, a prominent mucus barrier protein, was increased in the metformin-treated group compared to the DSS-treated group. Furthermore, fecal 16S rRNA analysis showed that metformin treatment changed the gut microbiota composition by increasing the relative abundance of *Lactobacillus* and *Akkermansia* species while decreasing *Erysipelatoclostridium* at the genus level. Antibiotic treatment partly abolished the anti-inflammatory and mucus-protecting effects of metformin. Administration of *A. muciniphila* alleviated the colonic inflammation and mucus barrier disruption. Metformin alleviated DSS-induced UC in mice and protected against cell damage *via* affecting the gut microbiota, thereby providing a new mechanism for the therapeutic effect of metformin in patients with UC. This study also provides evidence that *A. muciniphila* as a probiotic has potential benefits for UC.

## Introduction

Ulcerative colitis (UC), characterized by chronic and relapsing inflammation in the colon, is becoming a global disease (M’Koma, 2013). However, the underlying pathogenesis of UC is incompletely understood. It is thought to involve genetic factors, abnormal immune responses, environmental factors, intestinal barrier dysfunction, and dysbiosis (Zhang and Li, 2014; Testa et al., 2017; Ungaro et al., 2017). Goblet cell depletion and gut dysbiosis are important features of UC (Strugala et al., 2008; Gersemann et al., 2009; Johansson et al., 2014).

Mucus barrier dysfunction plays a significant role in the pathogenesis of UC. The mucus barrier, organized by the Mucin2 (Muc2) mucin, is a gel that covers the intestinal epithelium (Johansson and Hansson, 2011; Kühn and Klar, 2015). Several studies have shown that the thickness of the mucus is decreased in wild-type mice with DSS-induced UC, while Muc2-deficient mice developed spontaneous UC and were more susceptible to DSS-induced colitis (Van der Sluis et al., 2006; Heazlewood et al., 2008; Petersson et al., 2010). The mucus barrier is also impaired in patients with UC (Shen et al., 2018) (Van der Post et al., 2019).

The gut microbiota plays an important role in the pathogenesis of UC. The composition, diversity and richness of the gut microbiota are altered in patients with inflammatory bowel disease (IBD) (Knox et al., 2019) and are related to disease severity as well as treatment efficacy (Zhou et al., 2018). Furthermore, in animal models, the absence of gut microbiota reduces inflammation in the mouse colon (Hernández-Chirlaque et al., 2016). The manipulation of the microbiota by fecal microbiota transplantation (FMT) or administration of probiotics may have potential benefits in the treatment of IBD (Shen et al., 2014; Paramsothy et al., 2017).

Metformin, with a relatively low cost and a superior safety profile, is the first-line treatment for type 2 diabetes (McCreight et al., 2016). Recent studies have demonstrated that metformin has extensive benefits, besides its anti-diabetic effects, including anticancer effects (Leone et al., 2014), cardiovascular benefits (Rena and Lang, 2018; Van der Post et al., 2019), anti-aging effects (Anisimov, 2013), and anti-inflammatory effects (Saisho, 2015; Cameron Amy et al., 2016). In particular, metformin treatment increases the number of goblet cells in the intestine (Shin et al., 2014; Xue et al., 2016), indicating that metformin may have mucus protective effects. Several studies have established that metformin can attenuate colitis in mice by suppressing nuclear factor kappa-light-chain-enhancer of activated B cells (NF-kB) (Koh et al., 2014), Phosphorylated-Signal transducer and activator of transcription 3 (pSTAT3) (Lee et al., 2015), Jun N-terminal kinases (JNK) (Deng et al., 2018) and p38 mitogen-activated protein kinase (MAPK) activation (Di Fusco et al., 2018). However, most of these studies focus on the mechanisms involved in the host factor and the influence of gut microbiota on the therapeutic effects of metformin in colitis the is not fully understood.

Metformin can alter the gut microbiota by increasing the abundance of *Akkermansia muciniphila (A. muciniphila)* and *Lactobacillus* (Hur and Lee, 2015; Bauer et al., 2018; Zhang, 2019), both of which have an anti-inflammatory profile. Emerging evidence shows that *A. muciniphila* is decreased in inflammatory bowel diseases, which is negatively associated with inflammation scores (Png et al., 2010; Rajilić-Stojanović et al., 2013; Earley et al., 2019). *Lactobacillus* also shows potential benefits for IBD, as it has anti-inflammatory and gut barrier-protective effects (Vich Vila et al., 2018). The gut microbiota also has significant impacts on the mucus barrier: gut microbiota and their bacterial products regulate Muc2 secretion by goblet cells, and, hence, the properties of the mucus barrier (Petersson et al., 2010; Jakobsson et al., 2015; Birchenough et al., 2016). These studies suggest that gut microbiota altered by metformin may play a critical role in its anti-inflammatory effects. However, whether metformin exerts its anti-inflammatory and mucus barrier protective effects in UC *via* affecting the gut microbiota has not been determined.

Here, the present study proved that metformin exerted its anti-inflammatory and mucus-protective effects *via* affecting the gut microbiota, particularly, *via* enriching *A. muciniphila*.

## Methods

### Animal Model

All animal studies were carried out in specific pathogen-free (SPF) laboratory of the Department of Laboratory Animal, Southern Medical University, in accordance with the guidelines of the Southern Medical University Committee for Experimental Animals. And the protocols were approved by the Animal Experimental Ethics Committee of the Southern Medical University, China (**L2018225**). Wild-type male C57BL/6 mice (6–8-wk-old) ranging from 20 to 25 g in weight were purchased from the Animal Center of the Southern Medical University in China. The animals were housed at 22–24°C with an artificial 12 h/12 h day/night cycle in SPF conditions. Mice (6–8-wk-old) were treated with metformin (400 mg/kg/d), administered *via* oral gavage, 7 days before dextran sulfate sodium salt (DSS) treatment, and then in parallel with DSS treatment. Colitis in mice was induced as previously described (Chassaing et al., 2014). To be brief, DSS (MPbio, Solon, OH, United States) (2% w/v), with a molecular weight of 36,000–50,000 Da, was added to drinking water and administered to the mice for 6 days, followed by 1 day of regular water administration. The disease activity index (DAI) was evaluated after DSS administration as previously described (Wang et al., 2015). Fecal samples were collected on day 6 following DSS treatment. The mice were anesthetized by 100 mg/kg pentobarbital sodium (provide by the Department of Laboratory Animal, Nanfang Hospital) and euthanized by using cervical dislocation on day 7 after DSS treatment and the colons were removed.

### Depletion of the Gut Microbiota Using Antibiotics

The gut microbiota of mice was depleted according to a method described previously (Chen et al., 2020) with minor adjustments. Mice were administered with an antibiotic cocktail consisting of vancomycin 100 mg/kg/day, metronidazole 200 mg/kg/day, gentamicin 40 mg/kg/day, and ampicillin 200 mg/kg/day. The antibiotic cocktail was administered to the mice by oral gavage for 5 days prior to metformin treatment and the induction of experimental colitis described above. The fecal substance was collected on day 5 to evaluate the effect of the antibiotic treatment on the gut microbiota by using gel electrophoresis.

### Bacterial Culture and Animal Treatment

*Akkermansia muciniphila* bacteria (ATTC BAA-835), purchased from ATCC (Manassas, VA, United States), were cultured anaerobically (80% N2, 10% CO2, and 10% H2) at 37°C in brain heart infusion (BHI) medium (Zhai et al., 2019) with an AnoxomatTM MarkII anaerobic system (Mart Microbiology). Bacteria used for oral gavage were washed once using 5 ml of anaerobic phosphate-buffered saline (PBS, pH = 7.4) after centrifugation, and then resuspended in anaerobic PBS. The mice were orally gavaged with 1.50 × 10^11^ CFU bacterial cells per kilogram of body weight per day for 7 days prior to DSS treatment and 6 days parallel with DSS treatment. All the experiments were performed according to the General Requirements for Biosafety (GB 19489-2008).

### Histological Analysis

Tissues were embedded in paraffin and were cut into 4-μm sections and stained with hematoxylin and eosin (HE). The scores were assessed by two blinded investigators from the aspects of inflammation level, inflammation extent, crypt damage and involvement percentage according to the protocols devised by Deng et al. (Deng et al., 2018). Briefly, the level of inflammation was graded as (0) none; (1) slight; (2) moderate; (3) severe. The inflammation extent was graded as: (0) none; (1) mucosa; (2) mucosa and submucosa; (3) transmural. The crypt damage was graded on a scale from 0 to 4: (0) none; (1) basal 1/3 damaged; (2) basal 2/3 damaged; (3) only surface epithelium intact; (4) entire crypt and epithelium lost. These changes were quantified based on the percentage of disease process involvement as follows: (1) 1–25%; (2) 26–50%; (3) 51–75%; (4) 76–100% (Supplementary Table S1).

### Immunofluorescence

Immunofluorescence staining was performed as previously described with minor modifications (Robertson et al., 2008). Sections were deparaffinized in xylene three times for 10 min, and their endogenous peroxidase was quenched for 15 min using 3% hydrogen peroxide in methanol. Subsequently, the sections were rehydrated using a graded ethanol series and antigen retrieval was performed using a sodium citrate buffer at a sub-boiling temperature for 10 min. The slides were then cooled at the bench-top and washed with PBS three times for 5 min, blocked with 10% BSA for 1 h, and incubated overnight at 4°C with the following primary antibodies: anti-MUC2 (1:200, #MA5-12345; Invitrogen), anti-Kruppel-like factor 4 (KLF4) (1:100, #ab129473; Abcam), anti-mouse atonal homolog 1 (MATH1) (1:300, #PA5-29392; Abcam), anti-hairy and enhancer of split type-1 (Hes1) (1:200, #ab71559; Abcam), anti-SAM pointed domain-containing ETS transcription factor (SPDEF) (1:100, #LS-C749124; LSBIO). After washing three times for 5 min with PBS, the slides were incubated with the appropriate secondary antibodies conjugated with a fluorescent label for 1 h at room temperature in the dark. The sections were washed three times for 5 min in PBS and incubated with 4′,6-diamidino-2-phenylindole (DAPI) for 3 min at room temperature followed by PBS washing for another three times. Finally, the slides were mounted and investigated under a fluorescence microscope **(**BX53F; Olympus, Tokyo, Japan) using cellSens Dimension software.

### Alcian Blue-periodic Acid-Shiff (AB-PAS) Staining and Goblet Cell Counting

AB-PAS staining was performed using commercial kits (DG0007100; Solarbio, Beijing, China) according to the manufacturer’s manual. The number of goblet cells was determined by counting the Alcian Blue positive vacuoles (Becker et al., 2013).

### RNA Extraction

The total RNA from colon tissue was isolated using the Trizol method (Rio et al., 2010). Briefly, the colon samples were homogenized using 1 ml Trizol reagent per 80 mg of tissue. The homogenized samples were incubated for 10 min at room temperature. A total of 0.2 ml chloroform per 1 ml homogenized sample was used and the mixture was vortexed vigorously for 15 s. Then, the samples were incubated for 10 min at room temperature and centrifuged at 12,000 g for 10 min at 4°C. After centrifugation, the upper layer was transferred into a fresh tube. Then, 0.5 ml isopropyl alcohol was added to each tube and the samples were incubated for 10 min at room temperature. Next, the samples were centrifuged at 12,000 g for 10 min at 4°C and the supernatant was completely removed. A total of 1 ml of 75% ethanol was added per tube and the tube was washed gently. The samples were incubated for 10 min at room temperature and then centrifuged at 12,000 g for 10 min at 4°C. All the remaining ethanol was removed, the samples were air-dried and 20 μl diethylpyrocarbonate (DEPC) water was added per tube to dissolve the RNA.

### Real-time Quantitative PCR (RT-qPCR)

First-strand cDNA was synthesized using the PrimeScript RT Master Mix (Takara) according to the manufacturer’s manual, and RT-qPCR was performed using the Roche LightCycler 480II with the SYBR Premix Ex Taq. The primer sequences are provided in Supplementary Table S2. The 2^∆∆CT^ method was used to calculate the expression of the detected mRNA using β-actin as the reference gene (Deng et al., 2018).

### DNA Extraction

DNA was extracted from fecal samples by using the TIANamp Stool DNA Kit (#DP328; TIANGEN Biotech) according to the manufacturer’s instructions (Shao et al., 2017). The DNA was diluted to 1 ng/μl.

### PCR Amplification

Fecal DNA was amplified using the TIANGEN 2× Taq PCR MasterMix (KT201), according to the manufacturer’s manual and the V4 primers 514F (5′-GTGCCAGCMGCCGCGGTAA-3′) and 805R (5′-GGACTACHVGGGTWTCTAAT-3′) (Yin et al., 2015).

### 16S rRNA Sequencing

16S rRNA sequencing was performed as previously described (Janda and Abbott, 2007). Briefly, fecal DNA amplification was performed using the TIANGEN 2× Taq PCR MasterMix (KT201), according to the manufacturer’s manual. Amplification of the 16S/18S rRNA genes was performed using the specific primers with the barcodes: 16S V4: 515F-806R; 18S V4: 528F-706R; 18S V9: 1380F-1510R; ITS1: ITS5-1737F, ITS2-2043R; ITS2: ITS3-2024F, ITS4-2409R. Then, the PCR products were sequenced using the Illumina GAII (Illumina, San Diego, CA, United States) at the Beijing Genomic Institute (Shenzhen, China) and the data were clustered using the Illumina paired barcoded-sequencing (end) (BIPES) (PE) process for preliminary analysis. The rest of the sequences were screened by UCHIME and the suspected chimeric sequences were removed. All the reads were sorted into different samples according to their barcodes.

### Statistical Analysis

All the data from at least three independent experiments were displayed as the mean ± standard deviation (SD). The student’s t-test and/or one-way analysis of variance (ANOVA) with a proper post hoc Tukey-Kramer test comparison was carried out to determine if the differences were statistically significant. A *p* value of less than 0.05 was considered statistically significant.

## Results

### Metformin Alleviated DSS-induced UC in Mice

**FIGURE 1:**
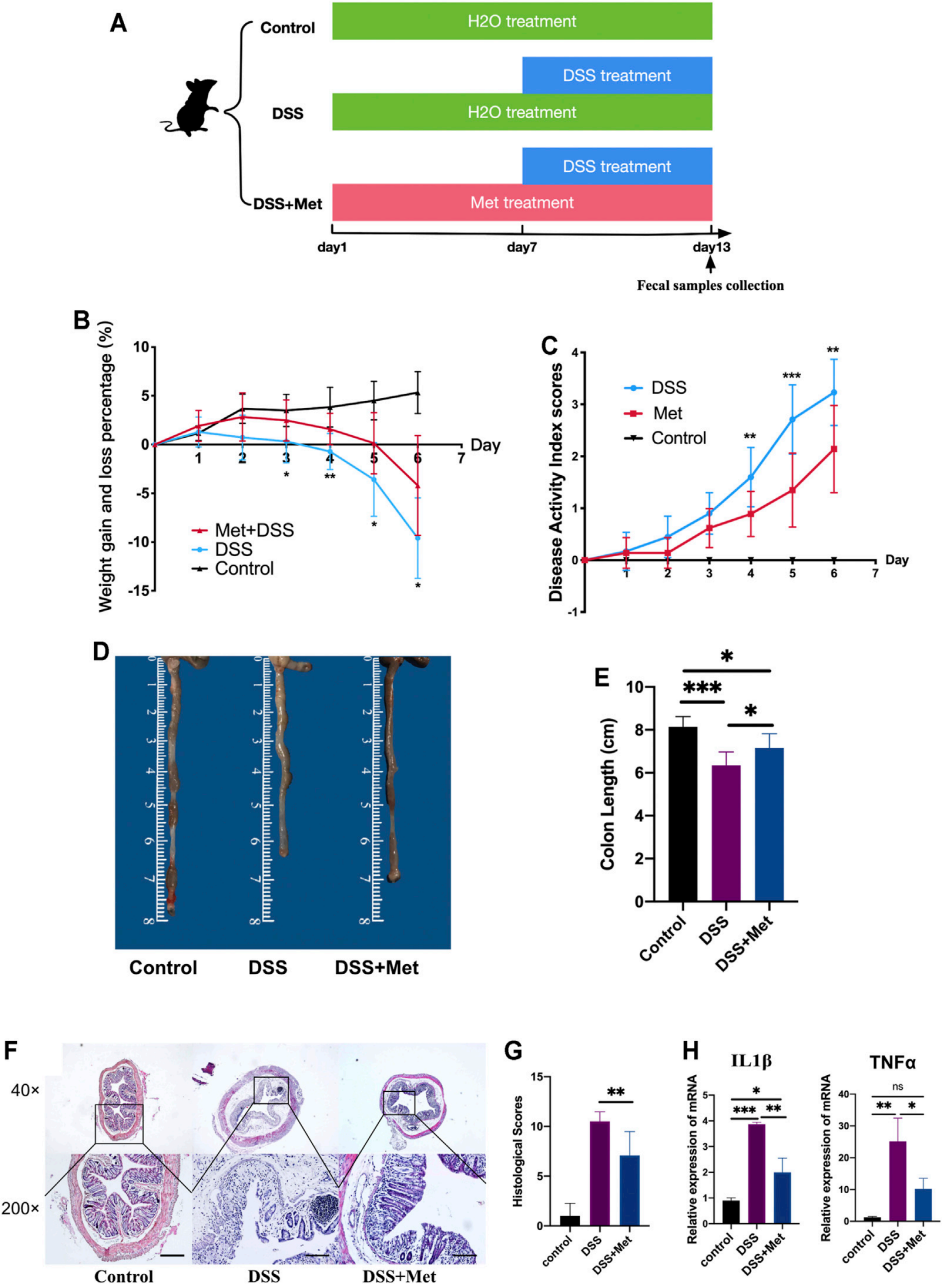
Metformin alleviates dextran sulfate sodium (DSS)-induced ulcerative colitis (UC) in mice. Flow chart of animal treatment (*n* = 6–10) **(A)**, weight loss and gain (*n* = 6–10) **(B)**, disease activity index (DAI) scores (*n* = 6–10) **(C)**, representative pictures of the mouse colon (*n* = 6–10) **(D)**, colon length (*n* = 6–10) **(E)**, hematoxylin and eosin (HE) staining (scale bars, 400 μm) (*n* = 6–10) **(F)**, and histological scores (*n* = 6–10) **(G)**. RT-qPCR assay of tumor necrosis factor-α (TNF-α) and interleukin 1β (IL1β) in colon tissue (*n* = 3) **(H)** and statistical comparison was made using an ANOVA test followed by Tukey-Kramer *post-hoc* tests. *p < 0.05, **p < 0.01, ***p < 0.001.

To determine if metformin can attenuate colitis, mice were first treated with 2% DSS to induce colitis and then treated with 400 mg/kg/day metformin or PBS solution (Figure 1A). Metformin reduced the severity of UC in mice as manifested by the reduced weight loss and less severe DAI scores (Figures 1B,C). Next, the colon length of mice was measured, and less colon length shortening was found in metformin-treated mice compared to mice treated with PBS (Figures 1D,E). Colon histology showed that metformin alleviated the damage and inflammation caused by DSS and reduced the histological scores (Figures 1F,G). The expression of pro-inflammatory cytokines interleukin 1β (IL1β) and tumor necrosis factor-α (TNF-α) in the colon were assessed. Consistent with previous results, metformin significantly reduced the level of IL1β and TNF-α (Figure 1H). These indicated that metformin had an anti-inflammatory effect.

### Metformin Protected Against Mucus Barrier Dysfunction in DSS-induced UC

**FIGURE 2:**
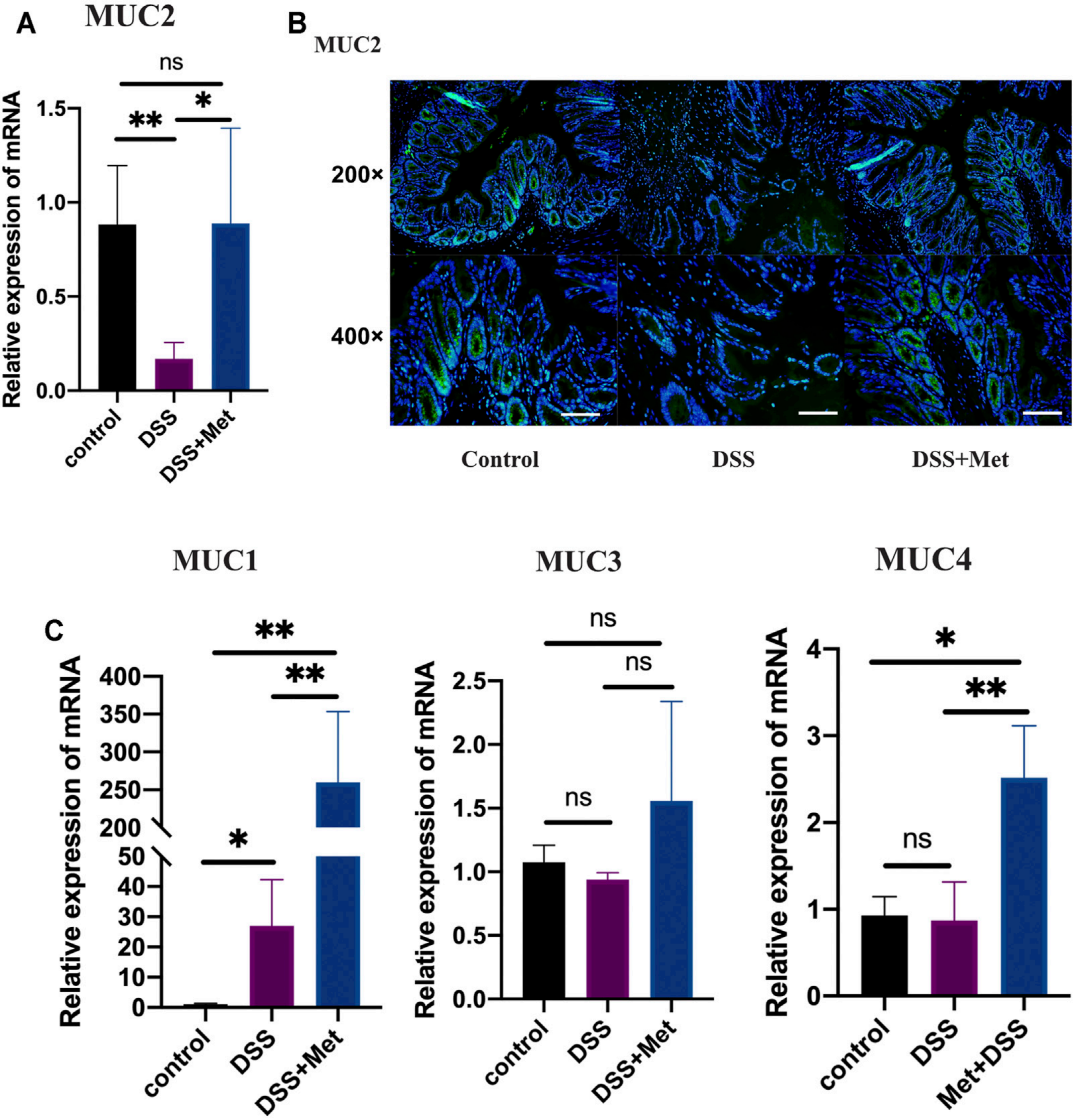
Metformin protects the mucus barrier by increasing the expression of mucins in ulcerative colitis (UC). RT-qPCR assay of mucin 2 (MUC2) expression of colon tissue (*n* = 3–4) **(A)**, immunofluorescence assay showing the expression of mucin2 protein in colon tissue (scale bars, 200 μm) (*n* = 4) **(B)**, and RT-qPCR assay (*n* = 3–6) of the relative expression levels of MUC1, MUC3, and MUC4 in colon tissue at mRNA level **(C)** and statistical comparison was made using an ANOVA test followed by Tukey-Kramer *post-hoc* tests. *p < 0.05, **p < 0.01, ***p < 0.001, ns, no significance.

Mucus barrier dysfunction plays an important role in the pathogenesis of UC. The present study has shown that metformin can attenuate UC in mice. We explored the effects of metformin on the mucus barrier of the mouse colon. The expression of MUC2, the major component of the mucus barrier, was increased in DSS-induced colitis at the mRNA level (Figure 2A). Additionally, immunofluorescence assays were performed to assess the expression level of mucin2 protein. Metformin treatment also increased mucin2 expression at the protein level (Figure 2B). Mucin1 (Muc1), mucin3 (Muc3), and mucin4 (Muc4) are critical components of the colon mucus barrier. Metformin treatment significantly increased the expression of Muc1 and Muc4 (Figure 2C). These results suggested that metformin can protect against mucus barrier dysfunction by increasing the expression of mucins, particularly that of mucin2, in the colon and thus contributed to its anti-inflammatory effect.

### Metformin Positively Regulated the Differentiation of Goblet Cells

**FIGURE 3:**
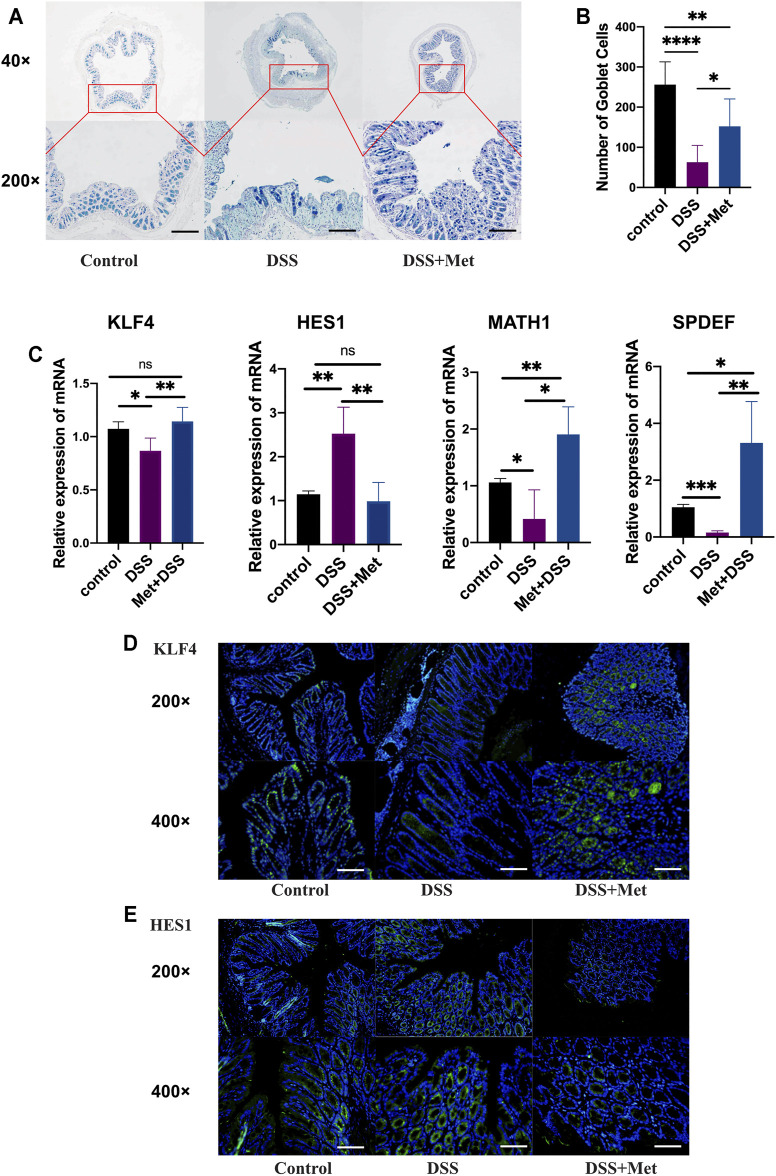
Metformin positively regulates the differentiation of goblet cells. Alcian blue-periodic acid-Shiff (AB-PAS) staining of colon tissue (scale bars, 400 μm) (*n* = 4–7) **(A)** and count of goblet cells (*n* = 4–7) **(B)**, RT-qPCR assay (*n* = 3–6) of Kruppel-like factor 4 (KLF4), hairy and enhancer of split type-1 (HES1), MATH1 and SAM pointed domain containing ETS transcription factor (SPDEF) at the mRNA level **(C)**, immunofluorescence assay of Klf4 (scale bars, 200 m) (*n* = 4) **(D)** and Hes1 (scale bars, 200 μm) (*n* = 4) **(E)** protein detection in the mouse colon and statistical comparison was made using an ANOVA test followed by Tukey-Kramer *post-hoc* tests. *p < 0.05, **p < 0.01, ***p < 0.001.

Mucin2 is synthesized and secreted by goblet cells. To explore the effects of metformin on goblet cells, the numbers of goblet cells in the colon were measured. Metformin treatment significantly increased the number of goblet cells (Figures 3A,B). KLF4, MATH1, SPDEF and HES1 play an important role in regulating the differentiation of goblet cells: KLF4, MATH1 and SPDEF can promote the differentiation of goblet cells while HES1 has an opposite effect. The expressions of them were assessed by RT-qPCR assays. Mice treated with metformin had a higher expression of KLF4, MATH1 and SPDEF and a lower expression of HES1 (Figure 3C). Furthermore, immunofluorescence assays showed that metformin treatment increased the expression of Klf4 and decreased that of Hes1 in colon tissue (Figures 3D,E), which suggested that metformin increased the number of goblet cells by promoting its differentiation.

### Metformin Changed the Structure of the Gut Microbiota in DSS-induced UC in Mice

**FIGURE 4:**
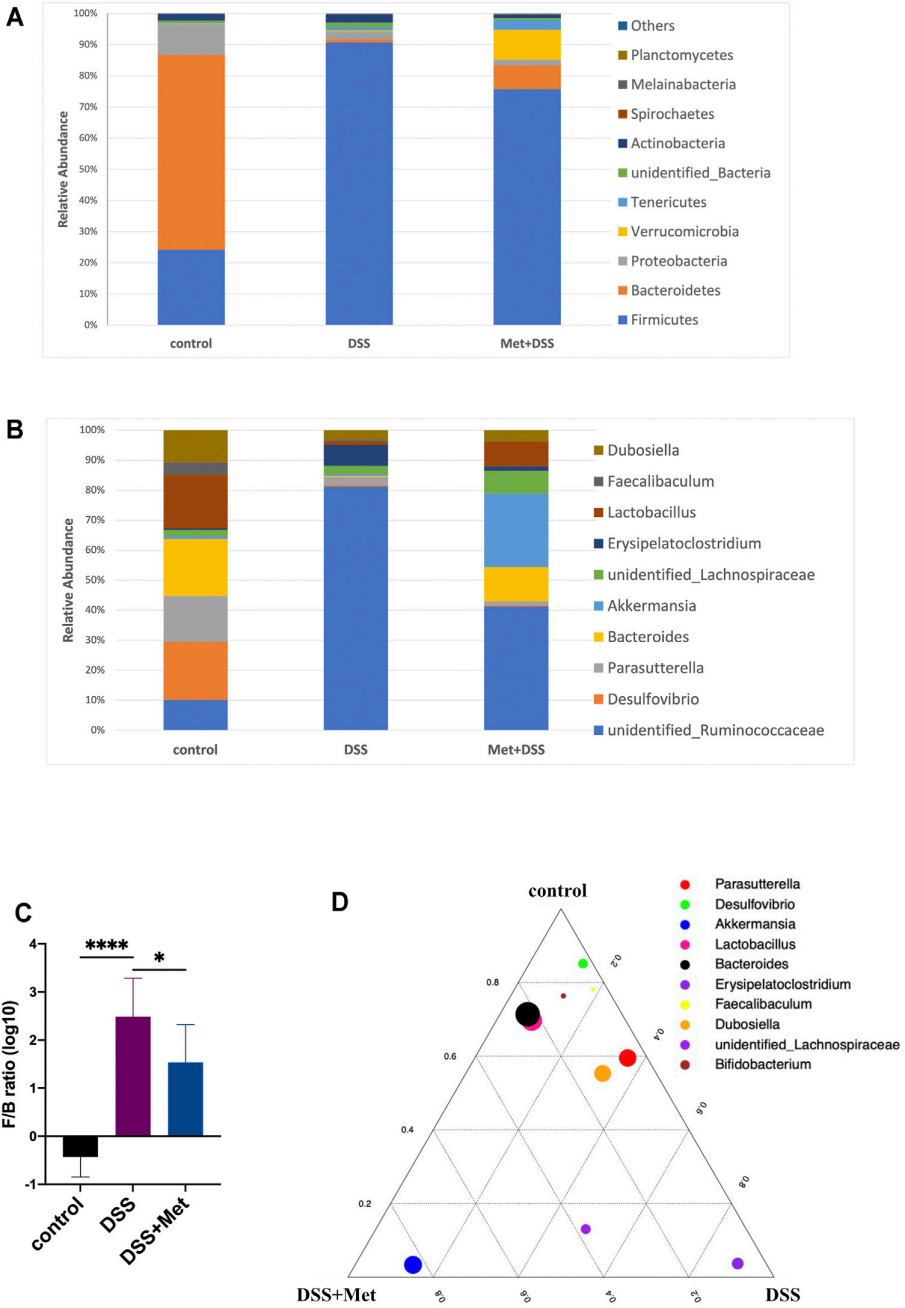
Metformin changes the structure of the gut microbiota (*n* = 4–10). Fecal 16S rRNA sequencing of gut microbiota of the metformin, dextran sulfate sodium (DSS) and control group. Relative abundance (% of the total community) of 10 most abundant phyla **(A)** and 10 most abundant genera **(B)**, Firmicutes/Bacteroidetes (F/B) ratio (log10 transformation) **(C)** and ternary plot indicating the fraction of 10 most abundant genera in the metformin group (bottom left corner), dextran sulfate sodium (DSS) group (bottom right corner) and control group (top corner) with the size of each dot representing the relative abundance **(D)**.

Previous studies have shown that metformin altered the structure of the gut microbiota of mice on a high-fat diet (Shin et al., 2014), which may play an important role in the anti-inflammatory and mucus-protective effects of metformin. 16S rRNA sequencing was performed to investigate the influence of metformin on the gut microbiota in DSS-induced UC in mice. At the phylum level, DSS treatment decreased the relative abundance of Bacteroidetes and Proteobacteria while it increased the relative abundance of Firmicutes compared with the normal control (Figure 4A). However, metformin treatment partly restored the structure of the gut microbiota in DSS-induced UC. The relative abundance of the phylum Bacteroidetes in the metformin group was increased, while the relative abundance of the phylum Firmicutes was decreased compared with the DSS group (Figure 4A). Furthermore, the relative abundance of the phylum Verrucomicrobia was increased after metformin treatment compared with the DSS and control groups (Figure 4A). At the genus level, metformin increased the abundance of *Bacteroides* and *Lactobacillus* compared with the DSS group. Metformin also increased the abundance of the genus *Akkermansia* compared with the DSS and control groups (Figure 4B). Increased or decreased Firmicutes/Bacteroidetes (F/B) ratio is regard as dysbiosis (Mariat et al., 2009). The F/B ratio increased by DSS treatment was lowered by metformin (Figure 4C), indicating that metformin reversed the dysbiosis in colitis mice. Importantly, the relative abundance of the genus *Akkermansia* was enriched in the metformin group (Figure 4D). These results showed that metformin can partly restore the structure of gut microbiota impaired by DSS treatment and enrich *Akkermansia* at the same time.

### Antibiotic Treatment Abolished the Anti-inflammatory Effects of Metformin

**FIGURE 5:**
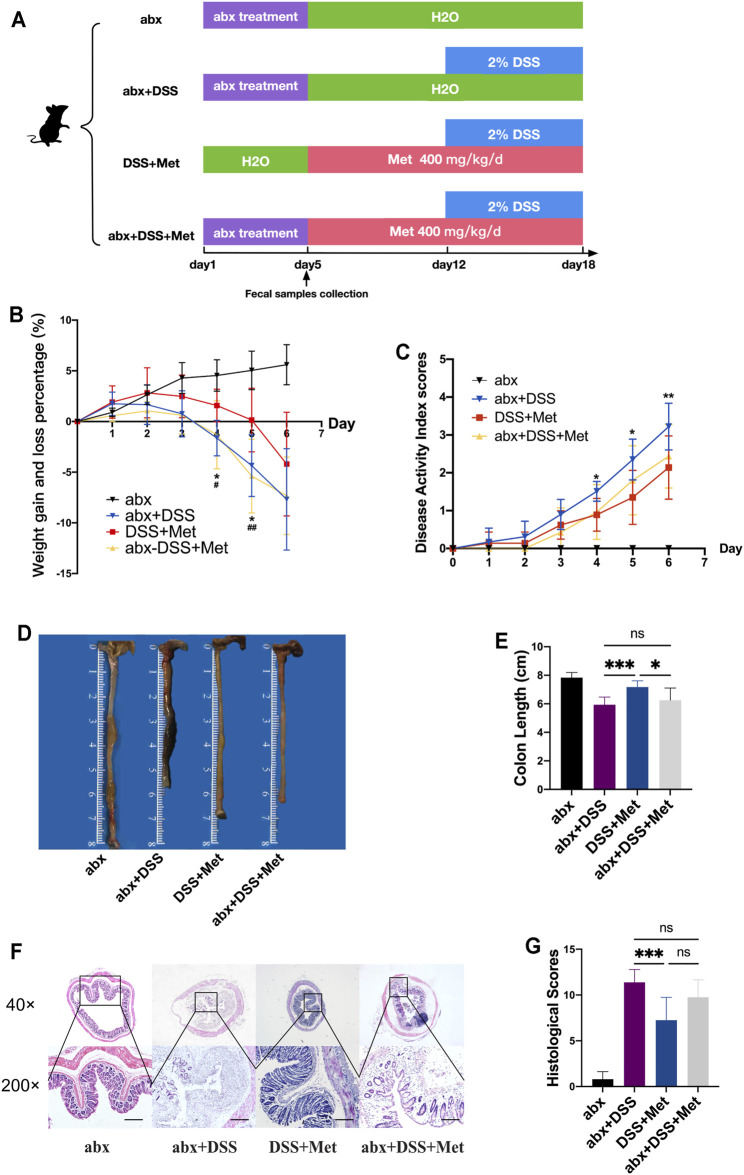
Antibiotics abolish the anti-inflammatory effects of metformin. Flow chart of animal treatment **(A)**, weight loss and gain (*n* = 6–10) **(B)**, disease activity index (DAI) scores (*n* = 6–10) **(C)**, representative pictures of the mouse colon (*n* = 6–10) **(D)**, colon length (*n* = 6–10) **(E)**, hematoxylin and eosin (HE) staining (scale bars, 400 μm) **(F)**, histological scores (*n* = 5–8) **(G)** *p < 0.05 (DSS+Met vs abx+DSS) and statistical comparison was made using an ANOVA test followed by Dunnett’s *post-hoc* tests. **p < 0.01, ***p < 0.00, ^**#**^ *p* < 0.05 (DSS+Met vs abx+DSS+Met), ^**##**^ *p* < 0.01.

To further investigate the effects of the gut microbiota on the anti-inflammatory beneficence of metformin, mice were treated with an antibiotic cocktail to deplete the gut microbiota (Figure 5A). The microbiota was depleted effectively after a 5-day antibiotic cocktail treatment (Supplementary Figure S1). There was no significant difference between the DSS and the antibiotics + DSS + metformin (abx + DSS + Met) group in weight loss and DAI (Figures 5B,C), which indicated that antibiotic treatment abolished the protective effects of metformin in DSS-induced UC. Mice in the abx + DSS + Met group also experienced more severe colon shortening compared with mice in the DSS + metformin (DSS + Met) group (Figures 5D,E). Histological analysis of colon tissue was performed for further verification. Although metformin inhibited the damage of colon tissue in DSS-induced UC, antibiotic treatment abolished these protective effects (Figures 5F,G), which suggested that gut microbiota is of great significance in the anti-inflammatory effects of metformin.

### Antibiotic Treatment Abolished the Mucus-protective Effects of Metformin

**FIGURE 6:**
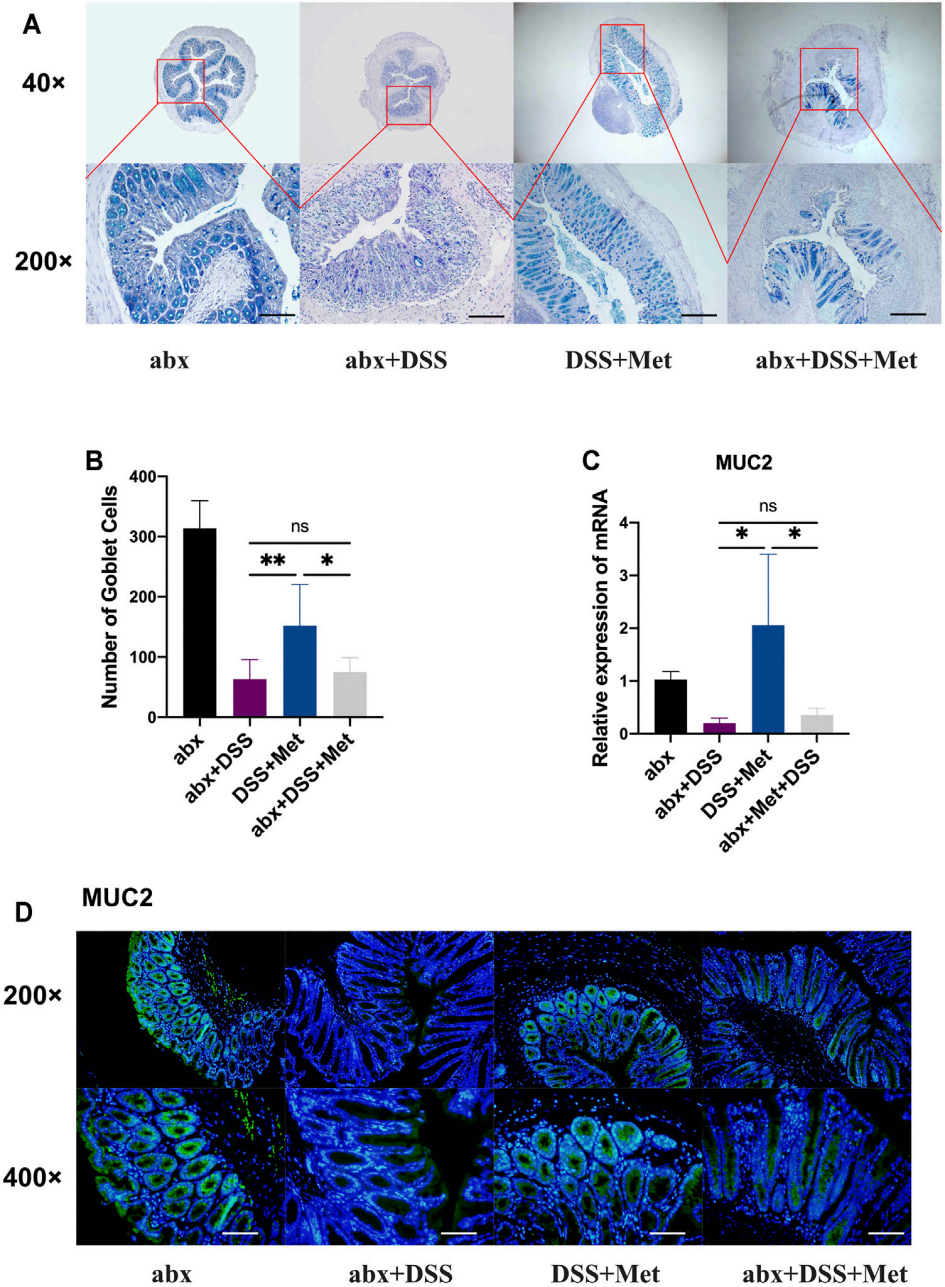
Antibiotics abolish the mucus-protective effects of metformin. Alcian blue-periodic acid-Shiff (AB-PAS) staining (scale bars, 400 μm) (*n* = 5–7) **(A)** and count of goblet cells (*n* = 5–7) **(B)**, relative expression levels of mucin 2 (MUC2) (*n* = 3–4) **(C)**, and immunofluorescence assay of mucin2 protein in the mouse colon (scale bars, 200 μm) (*n* = 3) **(D)** and statistical comparison was made using an ANOVA test followed by Dunnett’s *post-hoc* tests. *p < 0.05, **p < 0.01, ***p < 0.001.

Furthermore, the effects of the depletion of the gut microbiota on the mucus-protective effects of metformin were investigated by assessing the number of goblet cells and the expression of mucin2 at the mRNA and protein levels. The number of goblet cells in the DSS + Met group was increased compared with that of the DSS group while antibiotics treatment abolished such effects (Figures 6A,B). Antibiotics also inhibited the metformin-induced expression of MUC2 at the mRNA level and of mucin2 at the protein level (Figures 6C,D). The mucus-protective effect of metformin was also influenced by gut microbiota.

### *A. muciniphila* Exerted Anti-inflammatory and Mucus-protective Effects Similar to Those of Metformin

**FIGURE 7:**
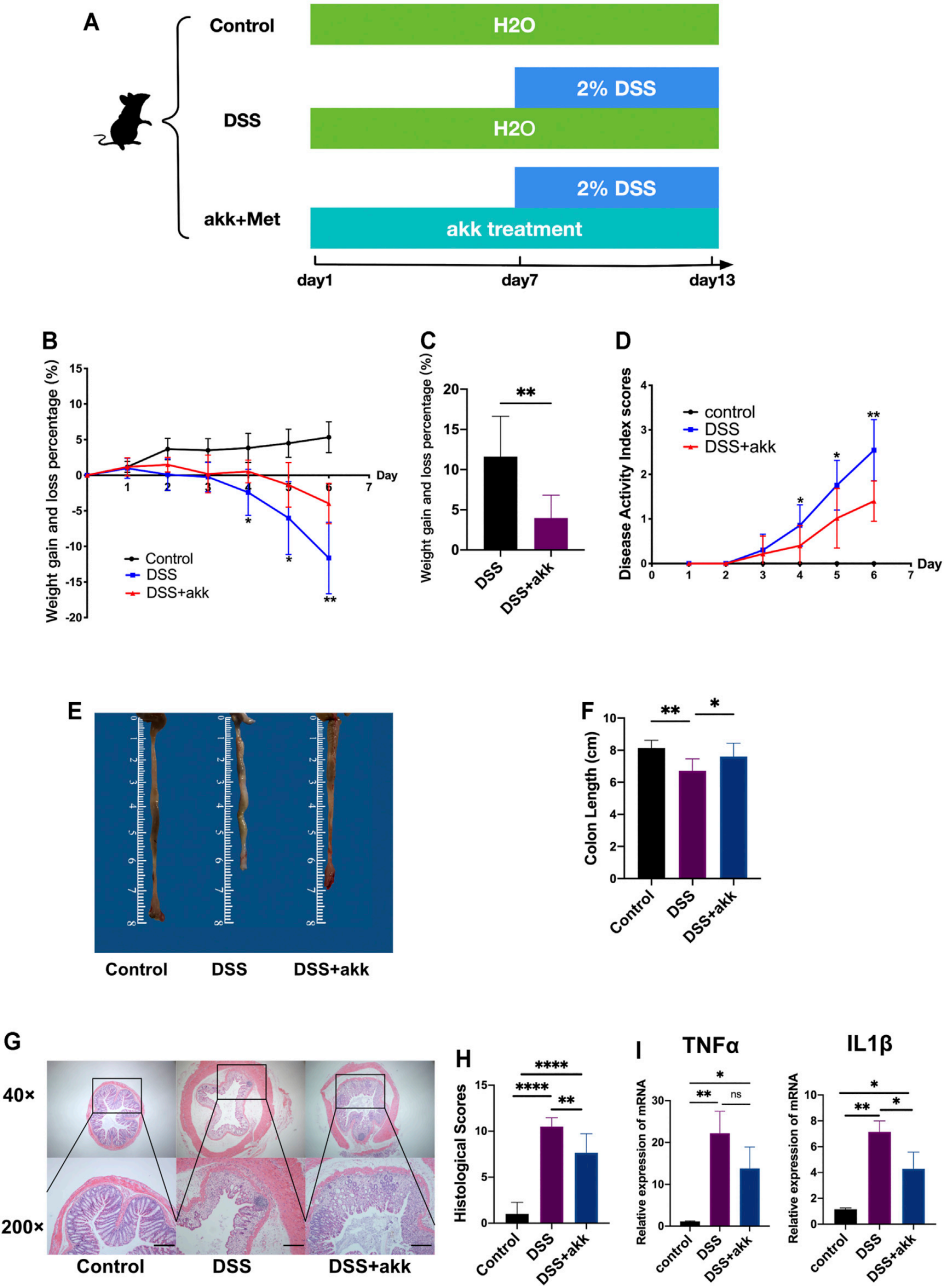
*Akkermansia muciniphila* alleviates dextran sulfate sodium (DSS)-induced ulcerative colitis (UC) in mice. Flow chart of animal treatment **(A)**, weight loss and gain during DSS treatment (*n* = 6–14) **(B)** and weight loss percentage at day 6 (*n* = 6–14) **(C)**, disease activity index (DAI) scores (*n* = 6–14) **(D)**, representative pictures of the mouse colon (*n* = 6–14) **(E)**, colon length (*n* = 6–14) **(F)**, hematoxylin and eosin (HE) staining of colon tissue (scale bars, 400 μm) (*n* = 6–10) **(G)**, colon histological scores (*n* = 6–10) **(H)**, and RT-qPCR assay of tumor necrosis factor-α (TNF-α) and interleukin 1β (IL1β) in colon tissue (*n* = 2–6) **(I)** and statistical comparison was made using an ANOVA test followed by Tukey-Kramer *post-hoc* tests. *p < 0.05, **p < 0.01, ***p < 0.001.

**FIGURE 8:**
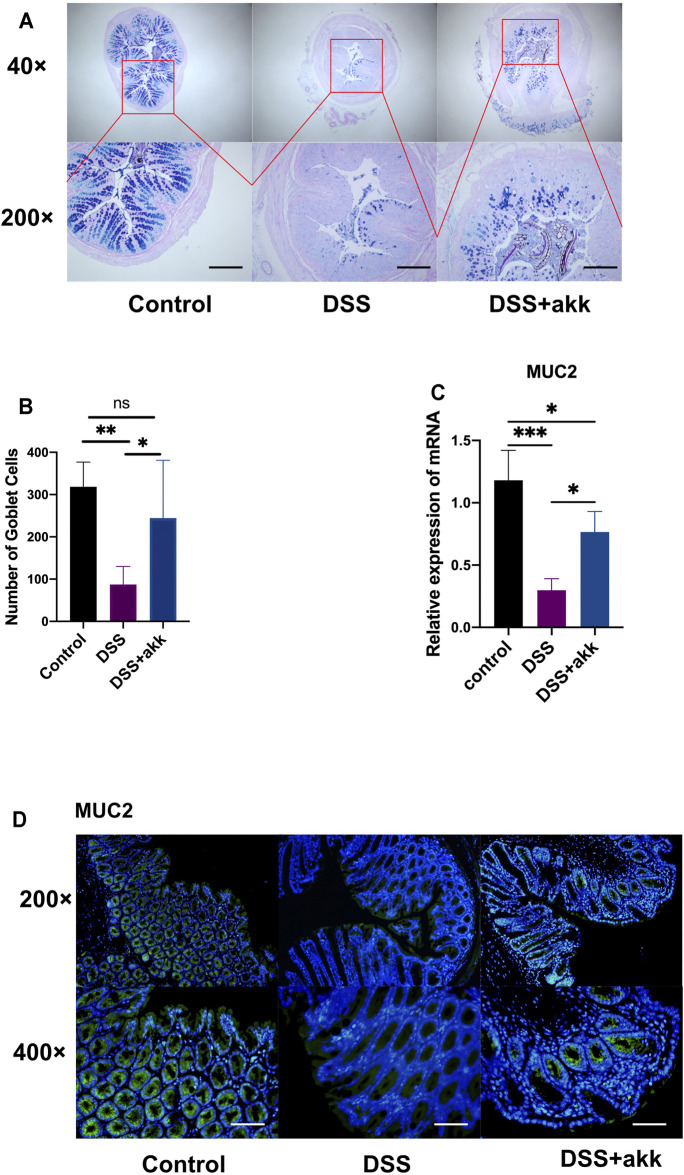
*Akkermansia muciniphila* protects the mucus barrier in ulcerative colitis (UC). Alcian blue-periodic acid-Shiff (AB-PAS) staining of colon tissue (scale bars, 400 μm) (*n* = 6–7) **(A)** and count of goblet cells (*n* = 6–7) **(B)**, RT-qPCR assay of mucin 2 (MUC2) (*n* = 3–4) **(C)**, and immunofluorescence assay of mucin2 protein detection in colon tissue (scale bars, 200 μm) (*n* = 4) **(D)** and statistical comparison was made using an ANOVA test followed by Tukey-Kramer *post-hoc* tests. *p < 0.05, **p < 0.01, ***p < 0.001, ns, no significance.

To explore whether *A. muciniphila* (akk)—which was enriched in the metformin group-exerts anti-inflammatory and mucus-barrier-protective effects like metformin, mice were administered with *A. muciniphila via* oral gavage (Figure 7A). *A. muciniphila* reduced weight loss (Figures 7B,C) and DAI scores (Figure 7D) in mice treated with DSS. Significant differences were found between the akk + DSS and DSS groups in colon length (Figures 7E,F) and histological scores (Figures 7G,H). Administration of *A. muciniphila* reduced the expression of TNF-α and IL1β, which were induced by DSS treatment (Figure 7I). In addition, *A. muciniphila* increased the number of goblet cells (Figures 8A,B) and the expression of mucin2 mucins (Figures 8C,D) compared with the DSS group, demonstrating that *A. muciniphila* has similar anti-inflammatory and mucus-protective effects.

## Discussion

The present study shows that metformin reduced colonic inflammation and protected against mucus barrier disruption along with an alteration of the gut microbiota during UC. Importantly, depletion of the gut microbiota partly abolished the anti-inflammatory and mucus-protective effects of metformin, indicating that metformin exerts its effects *via* the gut microbiota. Furthermore, administration of *A. muciniphila* alone, which was enriched in the metformin-treated group, to mice with DSS-induced UC mimicked the anti-inflammatory and mucus-protective effects of metformin. Based on these results the current study provides a critical understanding of the role of the gut microbiota in the anti-inflammatory and mucus-protective effects of metformin.

The results show that metformin mitigates the severity of mouse UC. Consistently, there is evidence showing that metformin exerts anti-inflammatory effects in mouse colitis: Di Fusco et al. reported that metformin reduced colonic inflammation by activating the AMP-activated protein kinase (AMPK) signaling pathway (Di Fusco et al., 2018); Lee et al. showed that metformin ameliorated colitis by suppressing STAT3 signaling *via* activating the AMPK signaling pathway (Lee et al., 2015). Deng et al. also has proved that metformin protected against intestinal barrier dysfunction *via* AMPKa1-dependent inhibition of JNK signaling activation and, thus, attenuated mouse colitis (Deng et al., 2018).

Accompanied by reduced colonic inflammation, metformin also restored the DSS-induced mucus barrier destruction by increasing the expression of mucin2, mucin1, mucin3, mucin4 and the number of goblet cells. Mucin1, mucin3 and mucin4 are transmembrane mucins. Their highly glycosylated extracellular domains can form a tight mesh structure and exert a barrier function. And their intracellular domains are involved in signaling pathways that regulate inflammation and cell differentiation (Putten and Strijbis, 2017). Mucin1 is upregulated in inflammation (Paone and Cani, 2020) which is consistent with the results of the present study. Mucin1 serves as a negative regulator of inflammation by suppressing the T helper 17-cell response (Nishida et al., 2012), toll-like receptor pathway and NLRP3 inflammasome complex activation (Dhar and McAuley, 2019), which may contribute to the anti-inflammatory effects of metformin in the current research. On the contrary, mucin3 and mucin4 are decreased during inflammation (Lindén et al., 2008). Mucin3 is negatively correlated with inflammation and dysbiosis levels (Algieri et al., 2021) *in vivo*, and can inhibit bacteria adherence *in vitro* (Pan et al., 2013). Kim et al. showed that mucin4 expression was decreased during DSS-induced colitis (Kim et al., 2019). However, contrary to Kim’s and results of the present research, Song et al. found that mucin3 was increased in the azoxymethane (AOM)/DSS colon cancer mouse model (Song et al., 2018). These may be due to the different experimental models that were used. Consistently, Xue et al. showed that metformin treatment increased the number of goblet cells by promoting secretory cell lineage differentiation, and reduced colonic inflammation *via* AMPK activation in IL10-knockout mice (Xue et al., 2016). Shin et al. also found that metformin treatment increased the number of goblet cells in mice fed a high-fat diet (Shin et al., 2014). In the clinic, goblet cell depletion and mucus barrier disruption were common in UC patients (Swidsinski et al., 2007; Strugala et al., 2008; Van der Post et al., 2019). Furthermore, the abnormality of the mucus barrier plays an important role in the pathogenesis of UC. Previous studies have shown that the aberrant mucus barrier structure caused by Muc2 deficiency led to a more severe colonic inflammation in a mouse colitis model (Van der Sluis et al., 2006; Heazlewood et al., 2008; Petersson et al., 2010). In this study metformin also promoted the differentiation of goblet cells by increasing the expression of KLF4, MATH1 and SPDEF and decreasing that of HES1. Wnt and Notch signaling play a central role in the regulation of intestinal epithelium differentiation (van der Flier and Clevers, 2009). More specifically, Wnt signaling promotes secretory cells differentiation (i.e., goblet, Paneth and enteroendocrine cells) and Notch signaling is required for enterocyte development. KLF4, MATH1 and SPDEF, which are stimulated by the Wnt pathway and inhibited by the Notch pathway, are critical for goblet cells formation (Gregorieff et al., 2009; van der Flier and Clevers, 2009). HES1, on the contrary, is promoted by Notch signaling and inhibits goblet cells development by decreasing the expression of MATH1 (van der Flier and Clevers, 2009). These findings indicate that the mucus barrier may have a critical protective effect against UC and metformin exerts a mucus-protective effect in DSS-induced colitis. Thus, we conclude that the mucus-barrier-protective effects of metformin may be attributed to its anti-inflammatory effects in the colon.

Metformin is a widely used anti-diabetic treatment. The interaction between metformin and the gut microbiota has been reported in previous studies. The concentrations of metformin in the intestine are much higher than those in the plasma (Bailey et al., 2008). Shin et al. showed that metformin exerted its pharmacological effects by modulating the gut microbiota in a diet-induced diabetes mouse model (Shin et al., 2014). Similar results have been reported by Bauer et al. (Bauer et al., 2018). However, most of these studies focus on diabetes mellitus. Although the gut microbiota plays a critical role in the pathogenesis of UC (Zhang and Li, 2014; Testa et al., 2017; Ungaro et al., 2017), its role in the pharmacological effects of metformin on UC remains unclear. In the current study, we showed that metformin changed the structure of the gut microbiota. At the phylum level, the alteration of the gut microbiota in IBD remains controversial. Santoru et al. showed that the levels of Firmicutes and Proteobacteria were increased in IBD, whereas those of Bacteroidetes were decreased (Santoru et al., 2017). In contrast, Frank et al. reported that the levels of Firmicutes and Bacteroidetes were decreased in IBD (Frank et al., 2007). Furthermore, Walker et al. showed that Firmicutes levels were decreased while those of Bacteroidetes were increased in IBD (Walker et al., 2011). Although the results of these studies are contradictory, they are to a certain extent consistent with our results. Importantly, metformin treatment partly restored the structure of the gut microbiota which was altered by DSS treatment at the phylum level. Furthermore, similar results were found at the genus level. Moreover, metformin treatment increased the relative abundance of the genera *Bacteroides*, *Lactobacillus* and *Akkermansia*, which are considered to be potential probiotics (Shin et al., 2014; Bauer et al., 2018; Tan et al., 2019). Accordingly, the F/B ratio increased by DSS treatment was restored by metformin. F/B ratio is widely accepted as a significant marker for intestinal homeostasis-increased or decreased F/B ratio is regarded as dysbiosis (Mariat et al., 2009). Previous studies have shown that the F/B ratio was increased in the DSS model and decreased when colitis was alleviated (Rodríguez-Nogales et al., 2018; Yeo et al., 2020). Taken together, these findings suggest that metformin may exert its anti-inflammatory and mucus-protective effects *via* modulating the gut microbiota. To confirm the hypothesis, we administered an antibiotic cocktail to mice *via* oral gavage to deplete the gut microbiota and the anti-inflammatory and mucus-protective effects of metformin were partly abrogated. The gut microbiota also has an important role in shaping the mucus barrier (Jakobsson et al., 2015). In germ-free rats, the structure of mucus in the colon is impaired and the thickness is reduced (Schroeder, 2019). Bergström et al. reported that compared with germ-free mice, the presence of the microbiota increased the expression level of Muc2 (Bergström et al., 2012). Moreover, Troll et al. reported that the microbiota promoted the differentiation of goblet cells by activating the Notch signaling pathway (Troll et al., 2018).

Notably, in the current study, *A. muciniphila* was enriched only in the metformin-treated group. *A. muciniphila* is a gram-negative, anaerobic bacterium (Derrien, 2004) which has been reported to be decreased in patients with UC (Earley et al., 2019). Shin et al. have shown that metformin increased the levels of *A. muciniphila* in mice on a high-fat diet, improved glucose homeostasis, and, interestingly, increased the number of goblet cells (Shin et al., 2014). The present study found that administration of *A. muciniphila* reduced the colonic inflammation and protected the mucus barrier disruption in UC. Bian et al. reported that the severity of DSS-induced colitis in mice was relieved upon administration of A. muciniphila (Bian et al., 2019), whereas Kang et al. reported that extracellular vesicles derived from A. muciniphila protected against DSS-induced colitis (Kang et al., 2013). These results further confirmed that metformin exerted its therapeutic effects on UC *via* modulating the gut microbiota and increasing the levels of potential probiotics (e.g. *A. muciniphila*).

The doses of metformin that used in this study are similar to those in patients receiving metformin treatment. In clinical practice, patients typically receive 500–2000 mg metformin per day (Scarpello and Howlett, 2008). According to a method based on body surface area normalization (Nair and Jacob, 2016), the human equivalent dose of 400 mg/kg/d in mice is 32.43 mg/kg/day or 1946 mg per day for a 60 kg adult, which is within the recommended range. Although lower doses of metformin may have fewer side effects, DSS-treated mice receiving 100 mg/kg/day metformin (equivalent to 486 mg per day for human) or 200 mg/kg/day metformin (equivalent to 972 mg per day for human) had more weight loss and more severe DAI scores compared to those treated with DSS and 400 mg/kg/day metformin (Supplementary Figure S2) in the current experimental settings. The current study chose the dose of metformin at 400 mg/kg/day. However, further study may be needed to investigate the effects of lower doses of metformin especially from the aspects of adverse events.

The limitations of the present study should be also mentioned. First, instead of germ-free mice, which are inaccessible to our facility, antibiotic-treated mice were used to explore the role of the gut microbiota in the effects of metformin on UC. Compared with germ-free mice, which is considered to be the golden standard for the study of microbiota, antibiotic-treated models may be more inconsistent. However, an antibiotic-treated mouse model is a less expensive and more accessible alternative that has been widely used by many researchers for the study of microbiota (Kennedy et al., 2018). Second, although significant differences were not observed between conventional mice and antibiotic-treated mice in the DSS-induced colitis model (data not shown), other potential unknown effects of antibiotic treatment on DSS-induced colitis were not taken into account in the present study. DSS-induced colitis model is very popular in IBD research because of its rapidity, reproducibility and similarities to human IBD (Solomon et al., 2010; Chassaing et al., 2014). But the exact mechanism through which DSS initiates colitis is unknown (Solomon et al., 2010). Previous studies have successfully developed DSS-induced colitis in germ-free and antibiotic-treated “pseudo-germ-free” mice (Hudcovic et al., 2001; Hernández-Chirlaque et al., 2016; Gancarcikova et al., 2020), indicating that gut microbiota may not be indispensable for the initiation of DSS-induced colitis. In addition, the manifestations of DSS-induced colitis in antibiotic-treated “pseudo-germ-free” mice are similar to those in conventional mice. Hernández-Chirlaque et al. showed that antibiotic-treated “pseudo-germ-free” mice had similar manifestations of DSS-induced colitis in aspects of weight loss, colonic inflammation and mucosal barrier function (Hernández-Chirlaque et al., 2016). Accordingly, Gancarcikova et al. revealed that antibiotic-treated “pseudo-germ-free” mice did not have changes that could influence the initiation or development of DSS-induced colitis, and the inflammatory process developed during DSS treatment was mostly due to the exposure to DSS and its toxic action on compactness and integrity of mucosal barrier (Gancarcikova et al., 2020). So, it is a feasible solution to use the antibiotic cocktail to explore the role of gut microbiota in the therapeutic effects of metformin in the DSS model. Third, except for *A. muciniphila*, the roles of other bacteria were not investigated, which may influence the pharmacological effects of metformin on colonic inflammation and the mucus barrier. The current study focused on the effects of *A. muciniphila* as it was enriched only in the metformin-treated group. However, these topics will be further investigated.

In summary, the results suggest that metformin alters the composition of the gut microbiota by increasing the levels of potential probiotics (e.g. *A. muciniphila*) and restoring the dysbiosis in mice with DSS-induced UC, which play a critical role in its anti-inflammatory and mucus-protective effects. The novelty of the current study is offering new insights into the role of the gut microbiota in the mucus-barrier-protective and anti-inflammatory effects of metformin, which indicates that metformin has the potential as a supplement or alternative to existing treatment for UC. It also provides evidence that A. muciniphila as a probiotic has potential benefits for the treatment of UC.

## Data Availability

The data presented in the study are deposited in the NCBI Bioproject, accession number PRJNA760645.
