# Supplementary material for: Metformin Exerts Anti-inflammatory and Mucus Barrier Protective Effects by Enriching Akkermansia muciniphila in Mice With Ulcerative Colitis
Source: Front Pharmacol. 2021 Sep 30;12:726707. doi: 10.3389/fphar.2021.726707 (PMC8514724; doi:10.3389/fphar.2021.726707)
Supplement: Supplementary file 1 [file Table1.docx]

| Feature | Grade | Description |
| --- | --- | --- |
| Inflammation level | 0 | None |
|  | 1 | Slight |
|  | 2 | Moderate |
|  | 3 | Severe |
| Inflammation extent | 0 | None |
|  | 1 | Mucosa |
|  | 2 | Mucosa and submucosa |
|  | 3 | Transmural |
| Crypt damage | 0 | None |
|  | 1 | Basal 1/3 damaged |
|  | 2 | Basal 2/3 damaged |
|  | 3 | Only surface epithelium intact |
|  | 4 | Entire crypt and epithelium lost |
| Involvement Percentage | 1 | 1–25% |
|  | 2 | 26–50% |
|  | 3 | 51–75% |
|  | 4 | 76–100% |

Table S1 Histological Analysis
