# Supplementary figures and images for: Metformin Exerts Anti-inflammatory and Mucus Barrier Protective Effects by Enriching Akkermansia muciniphila in Mice With Ulcerative Colitis
Source: Front Pharmacol. 2021 Sep 30;12:726707. doi: 10.3389/fphar.2021.726707 (PMC8514724; doi:10.3389/fphar.2021.726707)

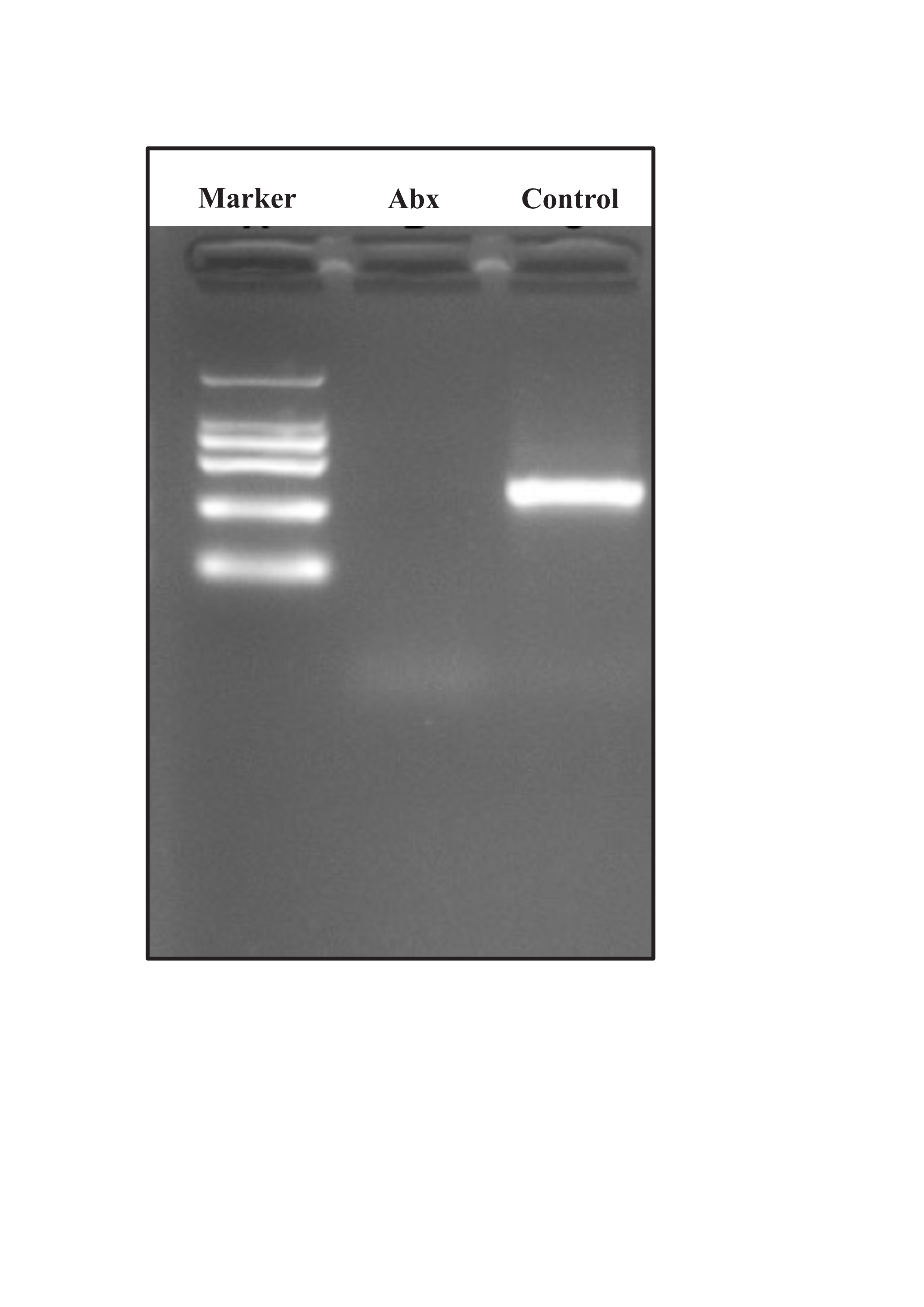

Supplement: Supplementary file 2 [file Image1.JPEG]

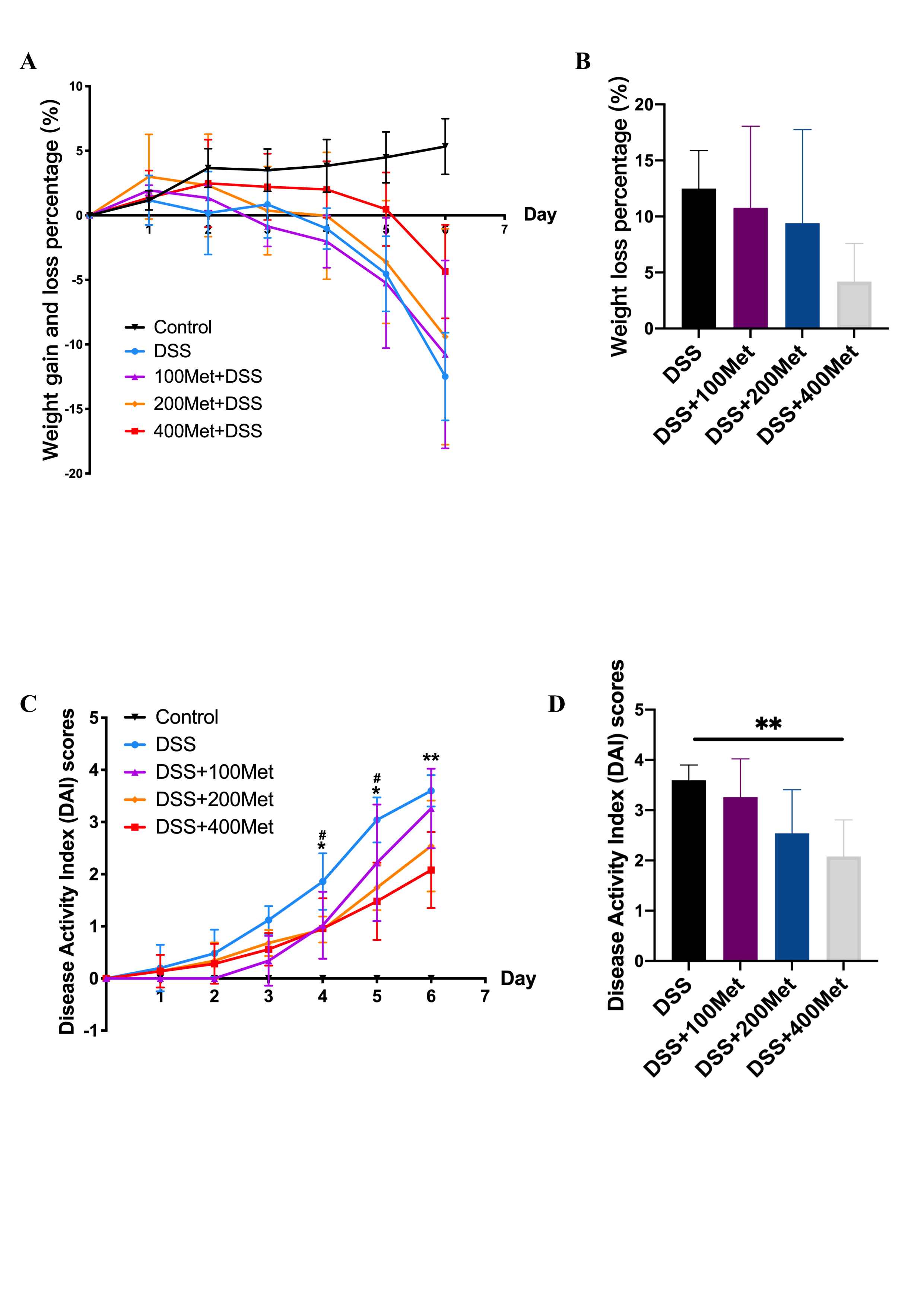

Supplement: Supplementary file 3 [file Image2.JPEG]
