# Supplementary material for: Metformin Exerts Anti-inflammatory and Mucus Barrier Protective Effects by Enriching Akkermansia muciniphila in Mice With Ulcerative Colitis
Source: Front Pharmacol. 2021 Sep 30;12:726707. doi: 10.3389/fphar.2021.726707 (PMC8514724; doi:10.3389/fphar.2021.726707)
Supplement: Supplementary file 4 [file Table2.docx]

Table. S2 Primer sequences used in RT-qPCT assays.

| **Gene** | **Forward** | **Reverse** |
| --- | --- | --- |
| Tnf-α | 5'-CAAAGGCGGAGATGAGACCC-3' | 5'-AGGCTTCTCCTTTGTGGTGAG-3' |
| Il1β | 5'-GATCCACACTCTCCAGCTGCA-3' | 5'-CAACCAACAAGTGATATTCTCCA TG-3' |
| Muc1 | 5'-GCAGCCTCAGGGCACCTC-3' | 5'-CACCGTGGGCTACTGGAGAG-3' |
| Muc2 | 5'-GCTGACGAGTGGTTGGTGAATG-3' | 5'-ATGAGGTGGCAGACAGGAGAC-3' |
| Muc3 | 5'-CGTGGTCAACTGCGAGAATGG-3' | 5'-CGGCTCTATCTCTACGCTCTCC-3' |
| Muc4 | 5'-CAGCAGCCAGTGGGGACAGCT-3' | 5'-CAGACACAGCCAGGGAACTC-3' |
| Hes1 | 5'-CCAGCCAGTGTCAACACGA-3' | 5'-AATGCCGGGAGCTATCTTTCT-3' |
| Math1 | 5'-GAGTGGGCTGAGGTAAAAGAGT-3' | 5'-GGTCGGTGCTATCCAGGAG-3' |
| Spdef | 5'-AAGGCAGCATCAGGAGCAATG-3' | 5'-CTGTCAATGACGGGACACTG-3' |
| KIf4 | 5'-GTGCCCCGACTAACCGTTG-3' | 5'-GTCGTTGAACTCCTCGGTCT-3' |
| βactin | 5'-AGAGGGAAATCGTGCGTGAC-3' | 5'-CAATAGTGATGACCTGGCCGT-3' |
